# Characterization of the complete mitochondrial genome of *Lepus comu*s, the endemic Lepus in China

**DOI:** 10.1080/23802359.2019.1624643

**Published:** 2019-07-11

**Authors:** Jing Huang, Wang Lin, Dandan Wu, Lifeng Cai, Xiumei Wang, Jianhui Huang

**Affiliations:** Fujian Provincial Key Laboratory of Ecology-Toxicological Effects & Control for Emerging Contaminants, Putian University, Putian; Key Laboratory of Ecological Environment and Information Atlas, Fujian Provincial University, Putian University, Putian; College of Environmental and Biological Engineering, Putian University, Putian, Fujian, China

**Keywords:** *Lepus comus*, *Lepus*, mitochondrial genome, phylogenetic relationship

## Abstract

*Lepus comu*s is the endemic species in China and has been listed on the IUCN Red List in 2013. In this study, we undertook and obtained the complete *Lepus comu*s mitochondrial genome. The circular mitochondrial genome sequence is 17,534 bp in size, containing 13 protein-coding genes (PCGs), 21 transfer RNA (tRNA) genes, 2 ribosome RNA (rRNA) genes and a longer D-loop region of 2,610 bp in length. The base composition of the mtDNA is as follows: 31.5% of A, 29.8% of T, 29.8% of C and 13.3% of G, with a total G + C content of the mtDNA 38.7% and A + T of 61.3%. The phylogenetic Maximum-Likelihood (ML) tree was constructed to validate the taxonomic status of *Lepus comu*s, exhibiting the closest relationship with *Lepus tibetanus*. The study of the complete *Lepus comu*s mitochondrial genome can provide a reference taxonomic relationships and more basic data for future.

*Lepus comu*s is the endemic species in China and has been listed on the IUCN Red List in 2013. *Lepus comu*s is called Yunnan hare and belongs to the genus Lepus, the family Leporidae and Lagomorpha. It is a mountainous species that occurs more frequently the Yunnan-Guizhou Plateau and was recognized as a valid species based on some specimens collected from Teng-chong, west of Yunnan Province, by Allen (Wu et al. 2000). *Lepus comus* only a minority of hares survive their first year in the wild, though survivors can reach 5 years, but in captivity it can live to 6 or 7 years. The remote habitat of this species is unlikely to be threatened, but increasing agricultural development in the valleys may isolate mountain populations (Yu et al. 2004). About the genome of *Lepus comu*s has not been published and few other studies, which makes it difficult to continue studying genetic evolution of this species. So, in this study, we obtained the complete mitochondrial genome of *Lepus comu*s, which contributes to a valuable and useful resource for the basic data for future.

The specimen sample of *Lepus comu*s was purchased from zoological garden in Putian district of Fujian province (Fujian, China, 119.00E; 25.44N). The total genomic DNA of *Lepus comu*s was extracted using the Animal Tissues Genomic DNA Extraction Kit and mtDNA stored in Putian University (No. PTU003). The whole genomic DNA was purified and fragmented using the NEB Next Ultra^TM^ II DNA Library Prep Kit (NEB, BJ, CN) that the mtDNA was sequenced. Quality control was performed to remove low-quality reads and adapters using the FastQC (Andrews 2015). The mitochondrial genome was assembled and annotated using the MitoZ (Meng et al. 2019). The physical map of the mitochondrial genome of *Lepus comu*s was generated using OrganellarGenomeDRAW (Lohse et al. 2013).

The complete mitochondrial genome of *Lepus comu*s (GenBank No. MK8324211) was a closed-circle genome in size of 17,534 bp, which coincided with many other vertebrate animals. The mtDNA of *Lepus comu*s comprised 36 genes, including 13 protein-coding genes (PCGs) (*NADH1-6, NADH4L, atp6, atp8, COX1-3* and *cytB*), 21 transfer RNA (tRNA) genes that is size from 57 bp (tRNA-Ala) to 69bp (tRNA-Leu), 2 ribosomal RNA (rRNA) genes (*12S rRNA* of 955 bp and *16S rRNA* of 1,578 bp) and a long D-loop region that is 2,160 bp in length. The base composition of the mtDNA is as follows: 31.5% of A, 29.8% of T, 25.4% of C and 13.3% of G, with a total G + C content of the mtDNA of 38.7% and A + T of 61.3%.

We used the complete mitochondrial genome of *Lepus comus* and 17 other species of the genus Lepus and Ochotona from GenBank to construct the phylogenetic Maximum-Likelihood (ML) tree. We used the RaxML v8 (Stamatakis 2014) to reconstruct the phylogenetic relationship (Figure 1) based on the Maximum-Likelihood (ML) method and used the best model (GTR + I + G), which the bootstrap value was calculated using 5,000 replicates to assess node support and all the nodes were inferred with strong support by the ML methods. The phylogenetic tree was constructed using the MEGA X (Kumar et al. 2018) and edited using the iTOL v4 (Ivica and Peer 2019). Analysis of phylogenetic relationship from the evolutionary tree results showed that *Lepus comus* is located in the genus Lepus and is closest related to *Lepus tibetanus* (LC073697.1). In this studies, *Lepus comus* is placed in the family Leporidae and can provide more basic data for the future research.

**Figure 1:**
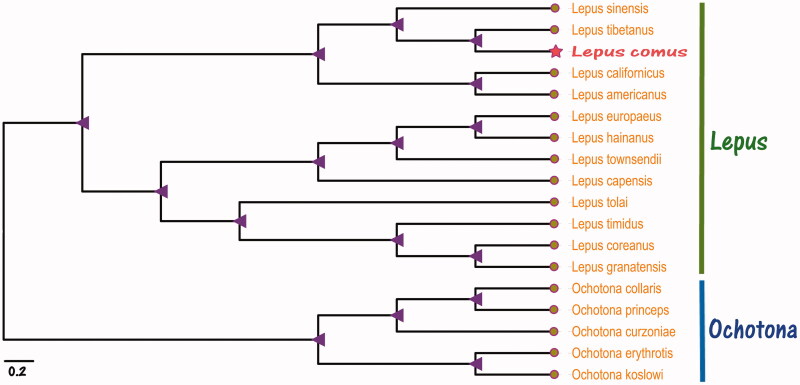
Maximum-Likelihood (ML) phylogenetic tree of the complete mitochondrial genome of *Lepus comus* and 12 other species in the genus Lepus and 5 other species in the genus Ochotona. The complete mitochondrial genome sequence of 17 species GenBank accession numbers follows: *Lepus americanus NC_024043.1, Lepus californicus KJ397614.1, Lepus capensis NC_015841.1, Lepus coreanus NC_024259.1, Lepus europaeus AJ421471.1, Lepus granatensis KJ397611.1, Lepus hainanus NC_025902.1, Lepus sinensis NC_025316.1, Lepus tibetanus LC073697.1, Lepus timidus KR019013.1, Lepus tolai NC_025748.1, Lepus townsendii NC_024041.1, Ochotona collaris NC_003033.1, Ochotona curzoniae NC_011029.1, Ochotona erythrotis NC_037186.1, Ochotona koslowi NC_039987.1, Ochotona princeps NC_005358.1.*
